# Chemical and cytotoxicity profiles of 11 pink pepper (*Schinus* spp.) samples via non-targeted hyphenated high-performance thin-layer chromatography

**DOI:** 10.1007/s11306-023-02008-8

**Published:** 2023-05-02

**Authors:** Fernanda L. B. Mügge, Gertrud E. Morlock

**Affiliations:** Chair of Food Science, Institute of Nutritional Science, and Interdisciplinary Research Center, IFZ, Justus Liebig University Giessen, Heinrich-Buff-Ring 26-32, 35392 Giessen, Germany

**Keywords:** *Schinus terebinthifolia*, *Schinus molle*, HPTLC, Bioprofiling, Triterpene, Bioassay

## Abstract

**Introduction:**

Pink pepper is a worldwide used spice that corresponds to the berries of two species, *Schinus terebinthifolia* Raddi or *S. molle* L. (Anacardiaceae). Toxic and allergic reactions by ingestion or contact with these plants were reported, and classical in vitro studies have highlighted the cytotoxic properties of apolar extracts from the fruits.

**Objectives:**

Perform a non-targeted screening of 11 pink pepper samples for the detection and identification of individual cytotoxic substances.

**Methods:**

After reversed-phase high-performance thin-layer chromatography (RP-HPTLC) separation of the extracts and multi-imaging (UV/Vis/FLD), cytotoxic compounds were detected by bioluminescence reduction from luciferase reporter cells (HEK 293 T-CMV-ELuc) applied directly on the adsorbent surface, followed by elution of detected cytotoxic substance into atmospheric-pressure chemical ionization high-resolution mass spectrometry (APCI-HRMS).

**Results:**

Separations for mid-polar and non-polar fruit extracts demonstrated the selectivity of the method to different substance classes. One cytotoxic substance zone was tentatively assigned as moronic acid, a pentacyclic triterpenoid acid.

**Conclusion:**

The developed non-targeted hyphenated RP-HPTLC–UV/Vis/FLD–bioluminescent cytotoxicity bioassay–FIA–APCI-HRMS method was successfully demonstrated for cytotoxicity screening (bioprofiling) and respective cytotoxin assignment.

**Supplementary Information:**

The online version contains supplementary material available at 10.1007/s11306-023-02008-8.

## Introduction

Plant species from the family Anacardiaceae are most commonly native to the tropical and subtropical regions, and comprise economically valuable plants that provide edible fruits and nuts, wood, and are used as ornamental species (Silva-Luz et al., 2022; Weeks et al., 2014). Well-known edible fruits are cashews (*Anacardium occidentale* L.) and mangos (*Mangifera indica* L.). The genus *Schinus* has also species used as ornamental in different regions of the world. Two of these species, native to South America, *Schinus terebinthifolia* Raddi and *S. molle* L., provide berries that are widely used as the spice pink pepper. Although not directly related to pepper (*Piper nigrum*) species, the pink pepper is appealing to consumers because of its bright pink color and mildly spicy flavor, as well as its useful properties for the conservation of food products (Giuffrida et al., 2020; Locali-Pereira et al., 2022).

These two *Schinus* species are commonly used in traditional medicine by local populations in Brazil and other South American countries for example to treat different types of inflammatory and rheumatic diseases. Historical records show that the most used parts of the trees are the bark, which is rich in oleoresin and has astringent and tonic properties, and the leaves, which contain essential oil. The fruits, used in less extent, also have oleoresins with a similar composition to the bark, and can also be used to extract essential oil (DATAPLAMT—Banco de Dados e Amostras de Plantas Aromaticas, Medicinais e Toxicas, 2023). The bark and fruits are used to treat urinary and uterine affections, bleeding and diarrhea, and rheumatic and respiratory diseases. Antioxidant (Bendaoud et al., 2010; Feriani et al., 2021; Kim et al., 2021; Oliveira et al., 2020b), antibacterial (D'Sousa' Costa et al., 2015; Linden et al., 2020; Salem et al., 2018), antifungal (Giordani et al., 2022), antidiabetic (Dos Santos da Rocha et al. 2019; Iwanaga et al., 2019), and other bioactivities have been reported. Anti-inflammatory properties, for example, have been linked to the composition of polyphenols, as well as monoterpenes and sesquiterpenes in the essential oil (Feriani et al., 2020; Formagio et al., 2011; Kim et al., 2021; Marangoni et al., 2022; Rosas et al., 2015; Yueqin et al., 2003).

In the 1980s, the toxicity or allergenic potential of pink pepper was brought to light, and the product was banned for sale in the United States of America by the Food and Drug Administration for a few years (Burros, 1982). Reports of severe anaphylactic reactions still happened after the ban was lifted, but the common hypothesis for these events was that allergic reactions occur predominantly due to cross-reactions in people that are allergic to other Anacardiaceae species such as cashew, pistachios, and mangos (Berghea et al., 2021; Fong et al., 2019). Some level of toxicity might also happen from previous sensitization with for example poison ivy or poison oak (*Toxicodendron* spp.), which are also from the same taxonomical family. Cardanol and urushiol are structurally similar irritant compounds from these plants (Symes & Dawson, 1953) that have also been described in the *S. terebinthifolia* berries, which are volatile and can also be found in the essential oil of the leaves (Kim et al., 2019; Stahl et al., 1983). Reports of toxicity, most remarkably for *Schinus terebinthifolia*, are also present in historical records, for people that have direct contact with the trees as well as for animals that ingest the leaves (for example cattle) or the fruits (especially birds) (DATAPLAMT—Banco de Dados e Amostras de Plantas Aromaticas, Medicinais e Toxicas, 2023).

Metabolomics is a powerful tool to discover new active molecules from traditionally used plant species, allowing non-targeted phytochemical analysis and with the assistance of network analysis and other computational approaches can help predict bioactivity (Makunga et al., 2022; Sharma & Yadav, 2022). The most commonly used profiling tools are nuclear magnetic resonance spectroscopy and various chromatographic techniques, such as LC–MS/MS and GC–MS/MS, followed by in silico analyses. High-performance thin-layer chromatography (HPTLC) has been successfully integrated into metabolomics workflows for quality control and authentication through fingerprinting and subsequent chemometric analysis such as principal component analysis (PCA) or hierarchical cluster analysis (HCA) (Booker et al., 2016; Ivanović et al., 2023; Mulaudzi et al., 2021; Salomé-Abarca et al., 2021). Hyphenated HPTLC and metabolomics are both excellent tools for simultaneous non-target bioprofiling of samples, whereby the investigation of the bioactivity is performed on-surface via HPTLC (Schreiner & Morlock, 2021; Schreiner et al., 2021) versus usually in silico via metabolomics. Both overcome laborious steps of bioactivity-guided fractionation of crude plant extracts (Kim et al., 2023).

The recently developed planar bioluminescent cytotoxicity bioassay can detect directly any cytotoxins present in the complex mixtures. The adherent luciferase reporter cells detect cytotoxic compounds as bioluminescence reduction from reporter cells (HEK 293 T-CMV-ELuc) applied directly on the reversed-phase (RP) HPTLC surface (Mügge & Morlock, 2022). The search for cytotoxic compounds is interesting in such samples in the context of discovering novel anti-cancer or anti-tumor agents, to improve selectivity towards tumor cells as well as to avoid resistance to commonly used therapies. For both *Schinus* species, previous works have shown that non-polar to mid-polar extracts obtained from the different plant parts (Garzoli et al., 2019; Mügge et al., 2021; Oliveira et al., 2018; Ovidi et al., 2021) and the essential oil obtained from the leaves (Díaz et al., 2008; Garzoli et al., 2019; Mahmoud et al., 2011; Santana et al., 2012) or fruits (Aboalhaija et al., 2019; Bendaoud et al., 2010; Guzzo da Silva et al., 2019; Matsuo et al., 2011; Oliveira et al., 2018) are active against different cancer cell lines. Most cytostatic or cytotoxic compounds used today in cancer treatment are derived from plants (Beutler, 2019; Davison & Brimble, 2019), but the advances in this field are slow due to the difficulty in applying high-throughput methods, which are useful for screening synthetic molecule libraries, to complex mixtures obtained from plant parts (Beutler, 2019; Davison & Brimble, 2019; Hackman et al., 2020; Huo et al., 2022; Mazumder et al., 2018; Najmi et al., 2022). The application of metabolomics in cancer research comes with many challenges, such as technological limitations, high costs of instrumentation, and data processing, among others (Oyenihi et al., 2021), but the introduction of non-targeted hyphenated HPTLC as a supplementary or supporting tool for metabolomic analysis can bring advantages for allowing both a pre-screening of interesting biological activities and direct determination of structure–activity relationship after the elution of active substances from the plate into HRMS or nuclear magnetic resonance (NMR) spectroscopy workflows (Ge et al., 2018, 2019).

This study, to the best of our knowledge, reports the first non-target bioprofiling intended to detect directly individual cytotoxic substances in *Schinus* berries. Interesting zones are eluted from the HPTLC chromatogram for flow injection analysis atmospheric-pressure chemical ionization high-resolution mass spectrometry (FIA–APCI-HRMS) recording and molecular formula assignment, saving time and costs.

## Materials and methods

### Chemicals and materials

HPTLC plates silica gel 60 RP-18 wettable (W) with (batch HX15025724) and without (batch HX28689296) fluorescence indicator F_254_, 20 cm × 10 cm, were obtained from Merck (Darmstadt, Germany). Bidistilled water was prepared using a Heraeus Destamat Bi-18E (Thermo Fisher Scientific, Schwerte, Germany). Solvents of high-performance liquid chromatography (HPLC) grade, including ethanol, methanol, ethyl acetate, *n*-hexane, tetrahydrofuran, acetone, toluene, Triton X-100, glycerol, methoxy benzaldehyde or* p*–anisaldehyde, vanillin, 2–aminoethyl diphenyl borate (natural product reagent A), sulfuric acid, and tris-(hydroxymethyl)-aminomethane (Tris) were obtained from Carl Roth (Karlsruhe, Germany). Acetic acid was purchased from VWR Chemicals (Radnor, PA, USA), and polyethylene glycol 6000 (PEG) was from J.T. Baker-Avantor (Deventer, Netherlands). Dulbecco’s Modified Eagle Medium (DMEM high glucose) with and without phenol red, fetal bovine serum (FBS), hygromycin B, and TrypLE Express solution were bought from Gibco (Carlsbad, CA, USA). HEK 293 T cells were obtained from the German Collection of Microorganisms and Cell Cultures (#ACC 635) and the generation of the genetically modified cell line HEK 293 T-CMV-ELuc is described elsewhere (Mügge & Morlock, 2022). Phosphate-buffered saline (PBS), ethylenediaminetetraacetic acid (EDTA), tricine, dithiothreitol (DTT), trans-1,2-cyclohexane diamine tetraacetic acid monohydrate (CDTA), citric acid, berberine chloride, all salts for buffer preparations, and penicillin/streptomycin solution for cell culture were from Sigma-Aldrich (Steinheim, Germany). Magnesium carbonate hydroxide pentahydrate was bought from Alfa Aesar (Karlsruhe, Germany). Double-concentrated PBS was obtained from Biochrom (Berlin, Germany). D-Luciferin sodium salt, adenosine triphosphate, oleanolic (OA), and ursolic acid (UA) were purchased from Cayman Chemical Company (Ann Arbor, MI, USA). Moronic acid (MA) was obtained from TCI Deutschland (Eschborn, Germany). Eleven samples of pink pepper (whole fruit) were bought in German supermarkets or online shops (Table S1).

### Plant extraction and standard solutions

Each sample was milled at 10,000 rpm for two rounds of 30 s using a small laboratory grinder (Tube-Mill control, IKA, Staufen, Germany). Two types of extracts were prepared for each sample, using either *n*-hexane or a mixture of water/ethanol/ethyl acetate (1/1/1, *V*/*V*/*V*). Each ground sample (300 mg) was placed inside a centrifuge tube and vortexed with 3 mL extractant for 30 s. The suspension was then ultrasonicated (Sonorex Digiplus, Bandelin, Berlin, Germany) for 30 min. After centrifugation at 3,000 × *g* for 10 min (Labofuge 400, Heraeus, Hanau, Germany), supernatants were transferred to sampler vials (100 mg/mL). Oleanolic (OA), ursolic acid (UA), and moronic acid (MA) standard solutions were prepared as 1 mg/mL solutions in methanol. Briefly, 2 mg of each substance were transferred to a 2 mL volumetric flask, dissolved, and filled up with methanol. An aliquot (1 mL) was transferred to a sampler vial.

### RP-HPTLC–UV/Vis/FLD method

After plate heating at 120 °C for 1 h (to fix the binder) and cooling down to room temperature, the plate was prewashed first with methanol and secondly with ethyl acetate, both up to 9 cm, each time followed by plate drying. Extracts were applied as 7-mm bands on the RP-18 W HPTLC plate (Automatic TLC Sampler 4, CAMAG, Muttenz, Switzerland). Plates were developed with either (1) toluene/ethyl acetate/methanol/acetic acid 30/5/2/1; or (2) water/acetonitrile/methanol/tetrahydrofuran/formic acid 15/5/5/1/1; or (3) *n*-hexane/acetone 4/1 (further mobile phase systems tested in Table S2). Plates were developed in a twin trough chamber (20 cm × 10 cm or 10 cm × 10 cm, CAMAG) up to 70 mm migration distance, measured from the lower plate edge, followed by drying in a stream of cold air (hair dryer) for 5 min. The developed plates were documented at Vis, UV 254 nm, and FLD 366 nm (TLC Visualizer 2, CAMAG). For derivatization, the following reagents were applied through automatic piezoelectric spraying (Derivatizer, CAMAG): *p-*anisaldehyde-sulfuric acid reagent (1 mL methoxy benzaldehyde, 140 mL methanol, 16 mL acetic acid and 8 mL sulfuric acid), vanillin sulfuric acid reagent (1 g vanillin, 80 mL ethanol and 0.8 mL sulfuric acid), natural product reagent A reagent (1 g 2–aminoethyl diphenyl borate in 100 mL ethanol), followed by a PEG solution (6% polyethylene glycol 6000 in ethanol), and berberine reagent (100 mg berberine chloride in 100 mL ethanol). The software visionCATS (version 3.1.21109.3, CAMAG) controlled the instruments.﻿

### HEK 293 T-CMV-ELuc cell culture

HEK 293 T cells stably expressing Enhanced Beetle Luciferase (ELuc) were obtained through transfection and selection with hygromycin B as described elsewhere (Mügge & Morlock, 2022). For the assays, cells were kept in a humified incubator at 37 °C and 5% CO_2_, and routinely sub-cultivated using DMEM supplemented with 10% FBS, 1 × penicillin/streptomycin, and 100 µg/mL of hygromycin. Upon reaching 80−90% confluence, cell monolayers were washed with PBS, dissociated from the flasks using TrypLE Express solution, and transferred to new flasks in a 1:3 to 1:6 dilution.

### Planar bioluminescent cytotoxicity bioassay

HEK 293 T-CMV-ELuc cells were harvested from the culture flasks, followed by cell counting using a Neubauer hemocytometer, and resuspension in assay medium DMEM/F12 without phenol red supplemented with 5% FBS solution and penicillin/streptomycin (Klingelhöfer et al., 2021; Mügge & Morlock, 2022). Due to the use of acid in some of the mobile phases, a two-step neutralization procedure (performed before cell application) improved cell survival and signal strength on the plates: (1) spraying 2 mL of 2.5% sodium bicarbonate solution (yellow nozzle, level 6, Derivatizer, CAMAG), followed by drying under a stream of cold air; (2) immersion in a citrate buffer solution of pH 12 (6 g/L citric acid monohydrate and 10 g/L of disodium hydrogen phosphate anhydrate), followed by plate drying (50 °C, TLC Plate Heater, CAMAG). Then, plates were immersed (immersion speed 3 cm/s, immersion time 5 s, Chromatogram Immersion Device, CAMAG) in double concentrated PBS (9.55 g in 500 mL of bidistilled water), and excess moisture was removed. Plates were placed inside a 3D printed 10 × 10 cm incubation chamber (Mügge & Morlock, 2022), and to avoid excessive moisture absorption from the cell tracks on the plate surface, 200 µL of a 10% glucose solution were pipetted above the solvent front. Cells were applied as a line (pipetted dropwise) on top of each sample track using 200 or 400 µL cell suspension (containing 5000 cells/µL). As a control, one or two additional parallel lines of cell suspension were applied on the plate background. After placing the glass cover on top of the incubation chamber, the chamber was sealed (using adhesive tape) for the 24-h incubation. For detection of the cell bioluminescence, the plate was completely dried under cold air (hair dryer), and immersed (immersion speed 3 cm/s, immersion time 5 s) twice into the luciferin detection solution (40 mM tricine, 2.14 mM magnesium carbonate hydroxide pentahydrate, 5.34 mM magnesium sulfate heptahydrate, 0.2 mM EDTA, 3 mM DTT, 1.1 mM D-luciferin and 20 mM adenosine triphosphate and mixed with lysis buffer containing 25 mM Tris pH 7.8, 2 mM DTT, 2 mM CDTA, 1% Triton X-100 and 10% glycerol, and citrate buffer pH 12). The HEK 293 T-CMV-ELuc cell bioluminescence was recorded using exposure times of 1 and then 10 min (Bioluminizer, CAMAG).

### RP-HPTLC−FIA-APCI-HRMS

The *n*-hexane extract of sample ID 4 was applied in triplicate (10 µL/band each) on a 10 × 10 cm RP-18 W F_254_ plate and separated using *n*-hexane/acetone (4/1, up to a migration distance of 70 mm), followed by solvent evaporation. Three cytotoxic substance zones were marked at UV 254 nm with a soft pencil, and eluted from the plate with ethyl acetate at a 0.2-mL/min flow rate for 90 s via the autoTLC–LC–MS interface (Mehl et al., 2021) into the same sampler vial. After evaporation of ethyl acetate under a stream of nitrogen, 200 µL methanol were added which were transferred to a conical insert vial. The elution was repeated for a blank plate background at a similar *hR*_F_ position as the target zone (also in triplicate on the same plate) to allow for the subtraction of the plate background from the analyte mass spectrum. Four microliters of each solution were injected (using methanol as eluent) for FIA-APCI^±^-HRMS via parallel reaction monitoring (Table S3). The instrument was controlled and the data were processed with Xcalibur 4.2.47 SP1 with Foundation 3.1.261.0 SP6 and SII for Xcalibur 1.5.0.10747 (Thermo Fisher Scientific).

## Results and discussion

### Chemical profiling of 11 pink pepper samples

It is well known that cultivation in different geographical locations and post-harvest processing can affect the chemical composition of plant products (Marangoni et al., 2022). Eleven commercial samples of pink pepper from various suppliers (Table S1) were studied to represent samples from various origins that might have undergone different cultivation, harvest, and post-harvest process to identify a common chemical profile for pink pepper. The extraction efficiency was studied using solvents or solvent mixtures with different selectivity. For the extraction of polar and mid-polar substances, methanol (I), ethanol (II), 90% ethanol in bidistilled water (III), and a mixture of ethyl acetate/ethanol/water in equal proportions (*V/V/V*) (IV) as well as, for the extraction of apolar substances, *n*-hexane (V) were used exemplarily for sample ID 1 (Fig. S1).

**Fig. 1:**
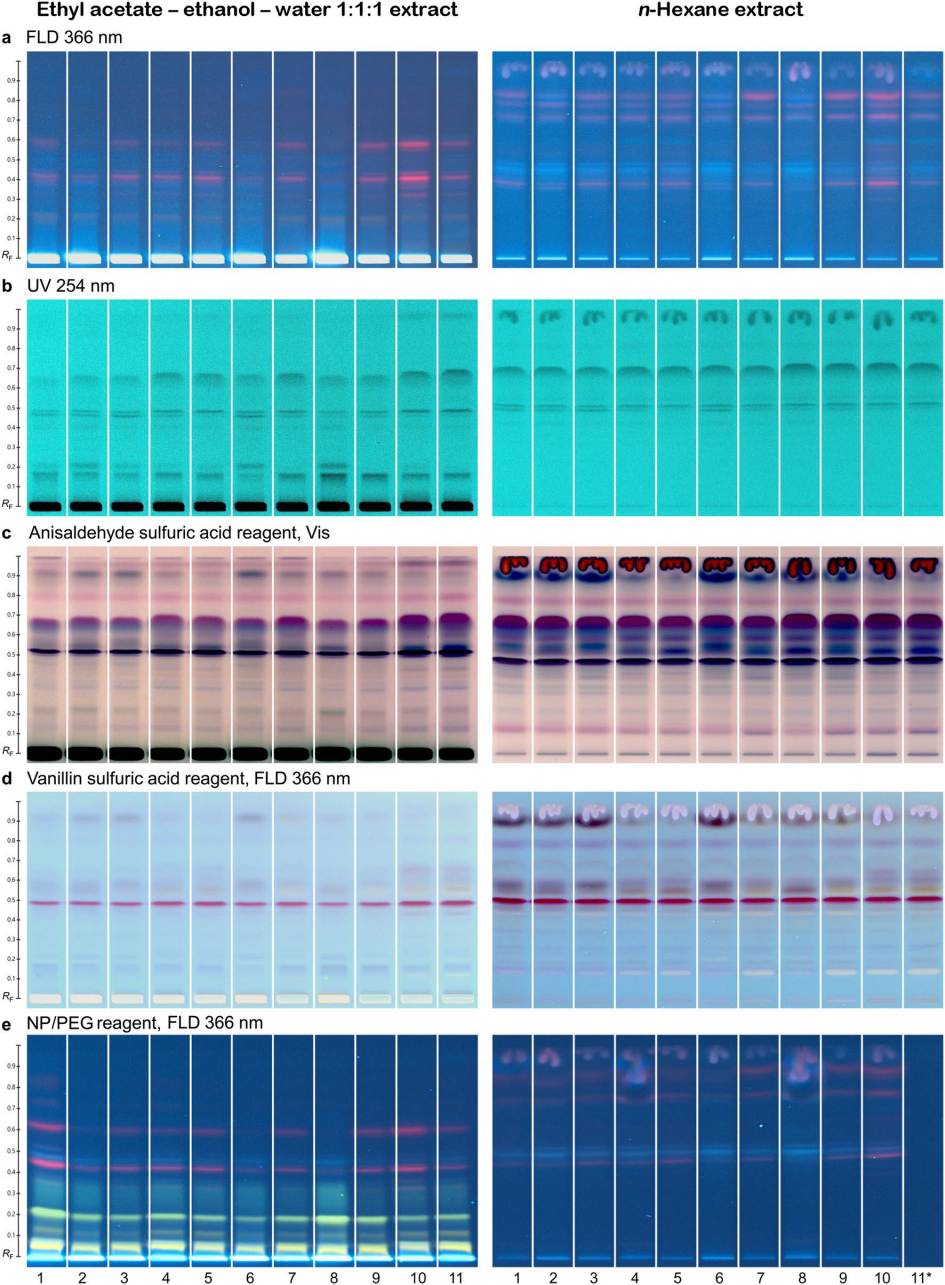
Physicochemical profiles of eleven commercial pink pepper samples. Extracts were prepared either with a mixture of ethyl acetate/ethanol/water 1/1/1 (*V/V/V*) or *n-*hexane, applied on RP-18 W HPTLC plates (10 µL/band, 100 mg/mL extracts, 7 mm bands), separated with toluene/ethyl acetate/methanol/acetic acid 30/5/2/1 (*V/V/V*) up to 7 cm, and detected at (**a**) FLD 366 nm, (**b**) UV 254 nm, (**c**) at white light illumination after derivatization with the *p-*anisaldehyde sulfuric acid reagent, (**d**) FLD 366 nm after the vanillin sulfuric acid reagent, and (**e**) FLD 366 nm after the natural product A reagent and polyethylene glycol 6000 (NP/PEG) (*sample ID 11 *n-*hexane extract was not applied due to an error)

Mobile phase development on RP-18 W plates (Table S2) using exemplarily the ethanol/ethyl acetate/water extract of ID 1 resulted in the mobile phase mixture toluene/ethyl acetate/methanol/acetic acid 30/5/2/1 (*V/V/V*﻿). Concomitant development of all five extracts showed their very similar composition when using a mid-polar mobile phase, whereby extractant IV showed a few additional bands compared to extractants I to III, while V showed more intense bands at *hR*_F_ values > 80 (Fig. S1). For having comparatively extracted more compounds than extractants I, II, and III, the extractant IV was chosen for the chemical profiling using multiple detection modes and was investigated in parallel with extractant V for all 11 samples. Ultraviolet light illumination and universal derivatization reagents (*p*-anisaldehyde and vanillin sulfuric acid reagents) were used to detect separated substances and reveal differences in composition between groups of extracts and among samples (Fig. 1a–d). Some prominent bands (*hR*_F_ 40–70) are common to all samples, although more intense using the extractant V. Most differences between both extractants were observed at the lowest and the highest *hR*_F_ range, which was expected. At *hR*_F_ ≥ 70, derivatization with the vanillin sulfuric acid reagent only revealed weaker bands in a few samples of extractant IV, while extractant V led to several bands. At *hR*_F_ < 23, absorbing bands at UV 254 nm were observed in the more polar extracts (ethyl acetate/ethanol/water), which also reacted with the natural product A reagent, followed by zone enhancement using polyethylene glycol 6000, to yellow fluorescent zones detected at FLD 366 nm (Fig. 1e). Due to the selective derivatization reagent, these bands were identified as phenolic compounds, such as flavonoids.

Another polar mobile phase system, consisting of water/acetonitrile/methanol/tetrahydrofuran/formic acid 15/5/5/1/1 (*V/V/V*) better separated the polar compounds evident via extractant IV after derivatization via the natural product A reagent and polyethylene glycol 6000 (Fig. S2; not present in the *n*-hexane extract, i.e. extractant V). In contrast, the apolar mobile phase system *n*-hexane – toluene – tetrahydrofuran 10:1:2 (*V*/*V*/*V*) separated very well the apolar sample part detected at white light illumination after derivatization with the *p*-anisaldehyde sulfuric acid reagent (Fig. S3). All 11 pink pepper extract samples had a very similar pattern and thus a similar compound composition at UV 254 nm and FLD 366 nm as well as after the respective derivatization reagent.

### Non-targeted cytotoxicity screening

**Fig. 2:**
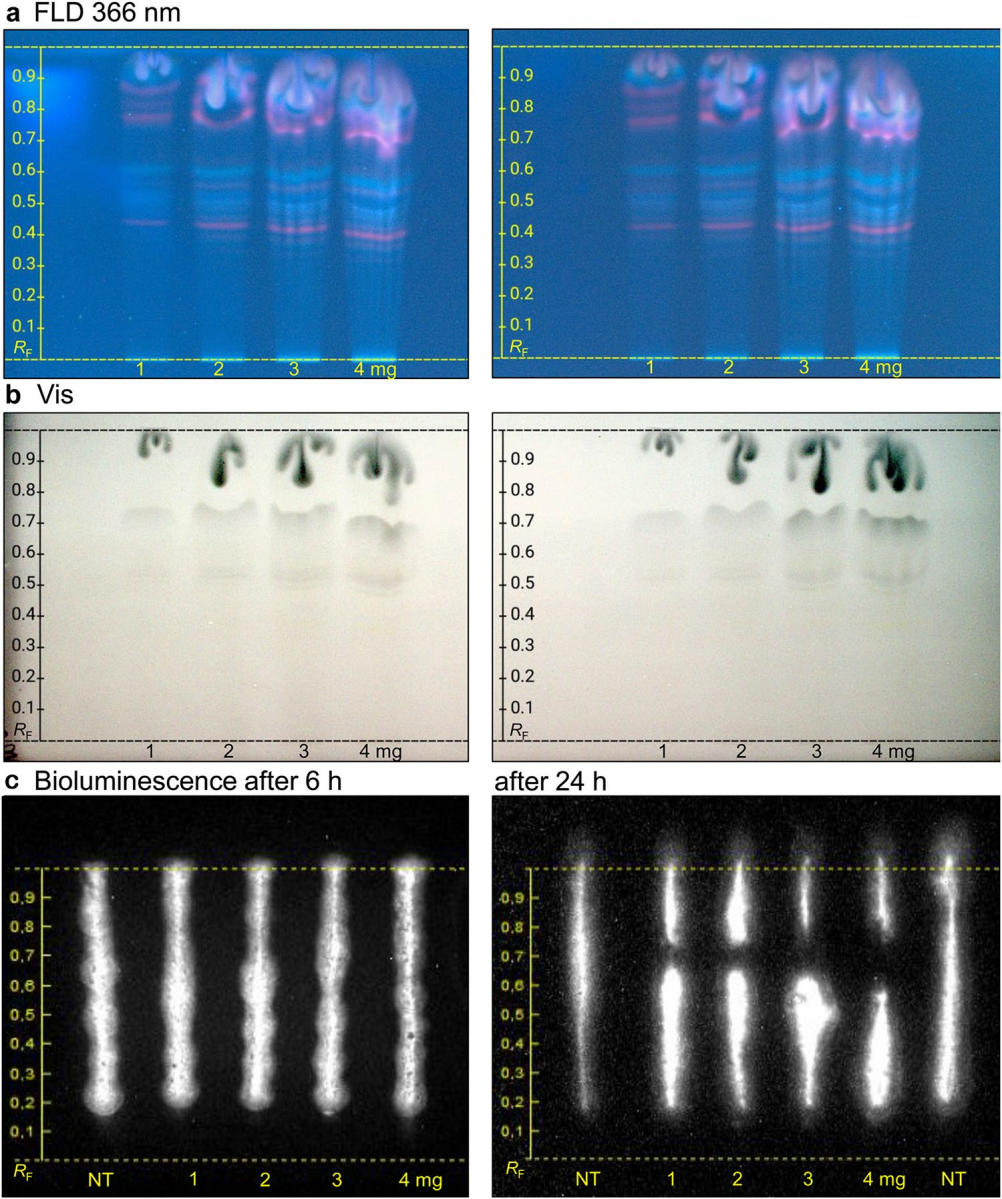
Cytotoxicity screening and dose–response dependency of the *n-*hexane extract of pink pepper sample ID 4 (100 mg/mL) analyzed as in Fig. 1 in increasing amounts (10, 20, 30, and 40 µL; 1, 2, 3, and 4 mg/band), and detected (**a**) at FLD 366 nm, (**b**) under white light illumination, and (**c**) via the bioluminescence (depicted as greyscale) of HEK 293 T-CMV-ELuc reporter cells incubated on the plate for 6 h or 24 h, dried for 5 min under cold air and immersed in lysis buffer containing D-luciferin; bioluminescence reduction indicates cytotoxic substance zones (NT: not treated cells used as negative control)

Four samples (IDs 4, 7, 8, and 9) were exemplarily analyzed using the polar mobile phase mixture water/acetonitrile/methanol/tetrahydrofuran/formic acid 15/5/5/1/1 (*V/V/V/V/V*) on the RP-18 W plate. In their obtained cytotoxicity profiles, any cytotoxic compound zone was not detected (Fig. S2 d), apart from a reduction of the bioluminescent cell stripe near the solvent front. Considering that extracts rich in phenolic compounds previously studied for *S. terebinthifolia* and *S. molle* are usually non-cytotoxic (Nocchi et al., 2016; Oliveira et al., 2020a) and that no significant difference in the compound pattern was observed between the 11 samples, further cytotoxicity screening was focused on the apolar mobile phase mixture toluene/ethyl acetate/methanol/acetic acid 30/5/2/1 (*V/V/V*﻿) and samples extracted with *n*-hexane. Exemplarily, sample ID 4 was applied at different volumes on the plate, representing 1−4 mg of extracted sample. After separation, it was evident that up to 20 µL (2 mg) sample extract could be analyzed without band distortion (Fig. 2a, b). Nevertheless, we used these plates for initial screening and proceeded with the 6-h on-surface incubation of the applied reporter cells as recently described (Mügge & Morlock, 2022). No cytotoxicity visualized as bioluminescence reduction was detectable after this 6-h period, with all cell tracks showing intense bioluminescence, even for the 4-mg sample (Fig. 2c).

In the classical in vitro methods used in previous studies to detect cytotoxicity, cells are usually incubated with samples for longer periods, ranging from 24 to 72 h. Therefore, we proceeded with a minimal adaptation of our method to allow for longer incubations. For that, instead of 200 µL cell suspension, 400 µL were applied per track, to minimize the loss of moisture from the cell track to the plate through adsorption of the assay medium as well as to allow for a more uniform distribution of cells and achieve stronger signals. Additionally, the glass plate covering the incubation chamber was tightened using adhesive tape to avoid moisture loss. With these adaptations, after a 24-h incubation, the applied cell strips appeared intact, and the plate had a normal amount of moisture. Not surprisingly, one cytotoxic substance zone at *hR*_F_ 70 was detected after the prolonged 24-h incubation period (Fig. 2c). The cytotoxicity was observed in all sample amounts tested (1−4 mg), and as expected, the area of the bioluminescence reduction observed was directly proportional to the concentration of the cytotoxic substance, as described also elsewhere (Mügge & Morlock, 2022).

**Fig. 3:**
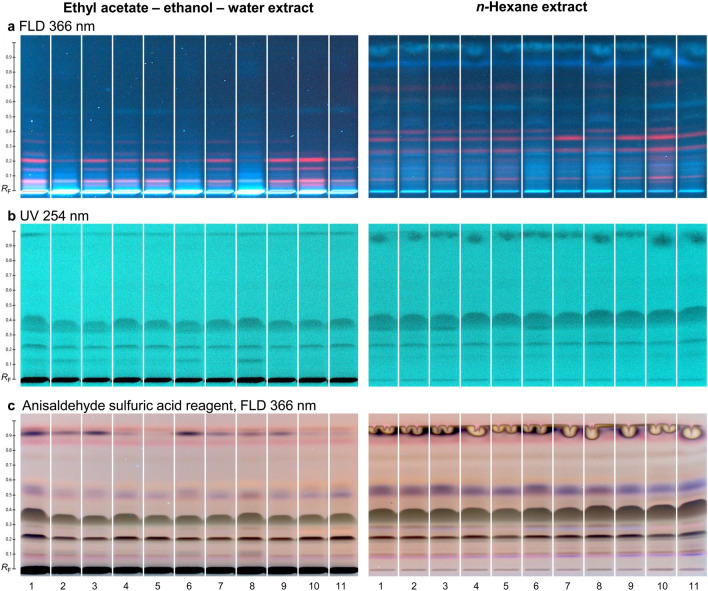
Physicochemical profiles of 11 commercially available pink pepper ethyl acetate/ethanol/water *versus n-*hexane extracts applied (10 µL/band, 100 mg/mL, 7 mm bands) on the RP-18 W HPTLC plate, separated with *n-*hexane/acetone 4/1 (*V/V*), and detected at (**a**) FLD 366 nm, (**b**) UV 254 nm, and (**c**) FLD 366 nm after derivatization with the *p-*anisaldehyde sulfuric acid reagent

To attempt a better separation and confirm the cytotoxicity results, a third even more apolar mobile phase, i.e. *n*-hexane/acetone 4/1 (*V/V*), was selected for the next experiments. The prominent cytotoxic band was now visible at *hR*_F_ 30−40 under UV 254 nm and after derivatization with the *p-*anisaldehyde sulfuric acid reagent (Fig. 3).

**Fig. 4:**
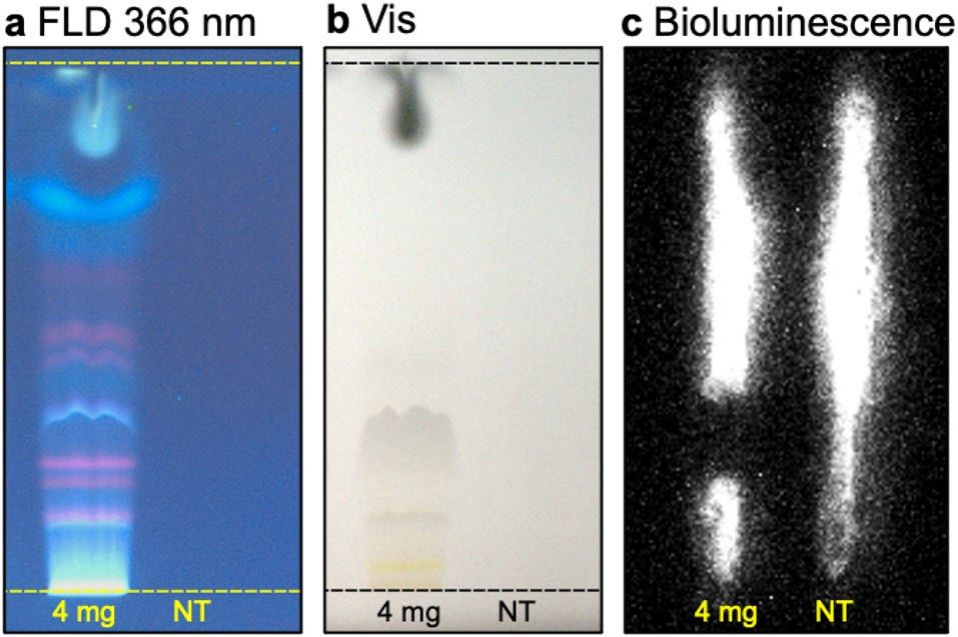
Confirmation of the cytotoxic zone in the *n-*hexane extract of sample ID 4 (40 µL, 7 mm band) analyzed as in Fig. 3 and detected at (**a**) FLD 366 nm, (**b**) white light illumination, and (**c**) via the bioluminescence reduction after incubation with HEK 293 T-CMV-ELuc reporter cells for 24 h (NT: not treated cells used as negative control)

After incubation with reporter cells for 24 h the cytotoxic activity of this band was confirmed (Fig. 4). This cytotoxic compound zone was present in all studied samples, so we decided to proceed with further investigation of this cytotoxic substance zone without discriminating differences between the profiles of each sample. It is noteworthy, however, that a longer incubation period of 48 or 72 h could highlight other cytotoxicity patterns or differences between samples, but at this point, the assay was not yet optimized for sterile conditions, and this was out of the scope of the present work.

### Identification of the cytotoxic substance

**Fig. 5:**
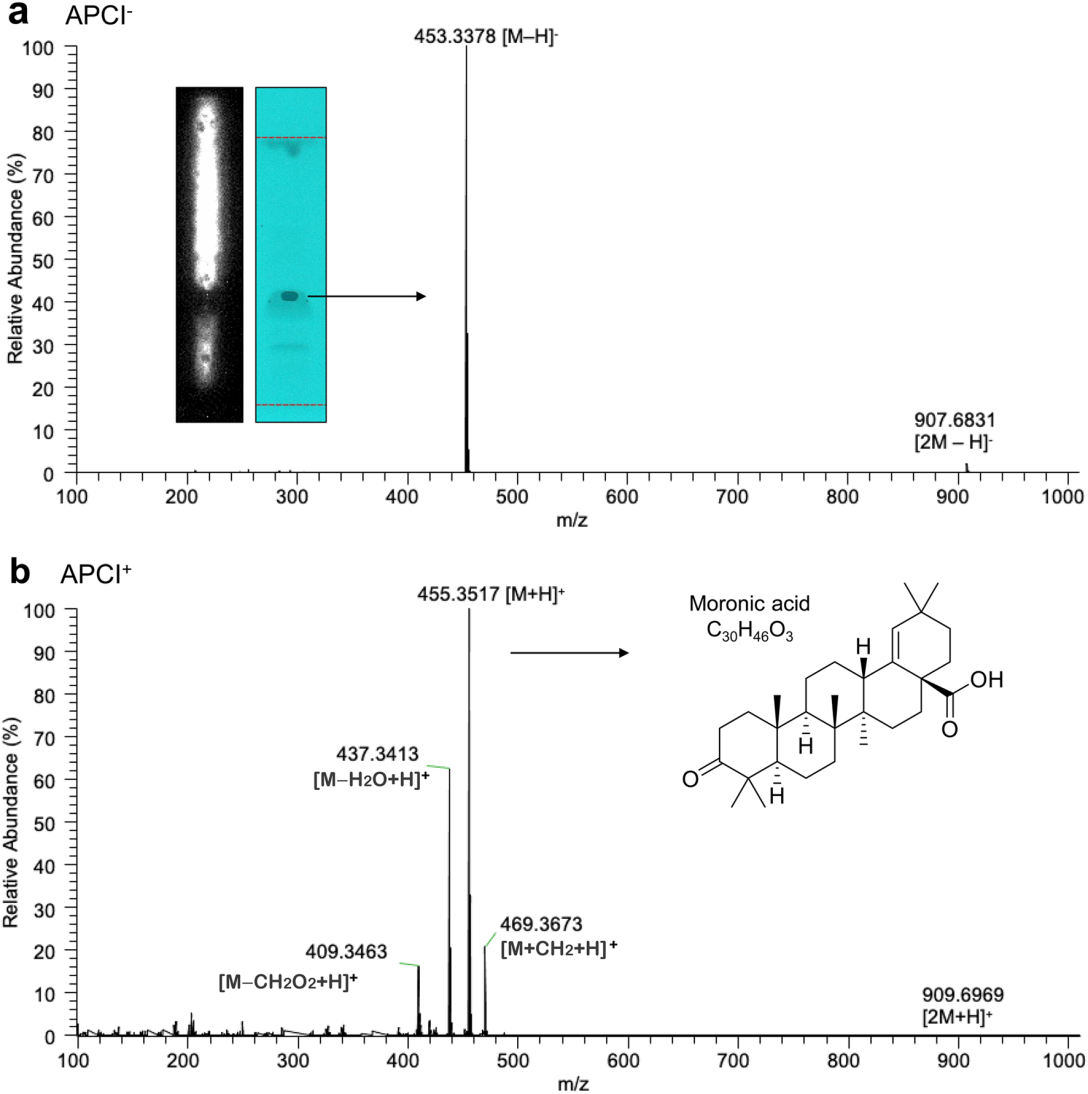
RP-HPTLC−FIA-APCI-HRMS spectra in the (**a**) negative and (**b**) positive ionization mode and respective signal assignments (Table S4) of the eluted cytotoxic substance zone in sample ID 4 analyzed as in Fig. 3 and tentatively assigned as moronic acid

In the chemical profiles obtained with *n*-hexane/acetone 4/1 (*V/V*) (Fig. 3), the cytotoxic substance zone at *hR*_F_ 30–40 showed UV absorbance and colorization via the *p*-anisaldehyde sulfuric acid reagent (Fig. 3b, c). For substance assignment, a new plate was applied with the *n*-hexane extract of sample ID 4 in triplicate. Each zone of interest (*hR*_F_ 30–﻿40﻿, detected at UV 254 nm) was eluted using ethyl acetate and pooled into the same sampler vial. After evaporation of the solvent, the residue was resuspended in methanol for better ionization in FIA−APCI-HRMS (Fig. S4). The APCI probe was chosen due to the low polarity of the substance. The HRMS spectrum obtained in the negative ionization mode showed the base peak signal at *m/z* 453.3378 [M-H]^−^, corresponding to the deprotonated molecule (Fig. 5a). The respective dimer was also observed. In the positive ionization mode, the signal at *m/z* 455.3517 [M + H]^+^ corresponded to the protonated molecule, while the signal at *m/z* 437.3413 [M-H_2_O + H]^+^ suggested the loss of water and the signal at *m/z* 409.3463 [M-CH_2_O_2﻿_ + H﻿]^+^ the loss of formic acid (Fig. 5b). Again, the respective dimer was also observed. These most abundant signals observed (Table S4, all signal assignments showed a mass error < 0.60) corresponded to the mass and fragmentation patterns of a triterpenoic acid with the molecular formula of C_30_H_46_O_3_ tentatively assigned as moronic acid (Rhourri-Frih et al., 2009; Sut et al., 2018; Vahur et al., 2012). Several triterpenoic acids have been discovered in *Schinus* fruits or isolated seeds when extracted with apolar solvents such as *n*-hexane and dichloromethane (Kaistha & Kier, 1962; Vieira et al., 2015), but not always showed cytotoxic activity. The signal at *m*/*z* 469.3673 was assigned to the molecular formula C_31_H_48_O_3_ (mass error 0.68 ppm) which was explained by its methyl ester formation. During aging oxidation of the C_30_H_46_O_3_ molecule could occur, however, was not observed here (van der Doelen et al., 1998).

**Fig. 6:**
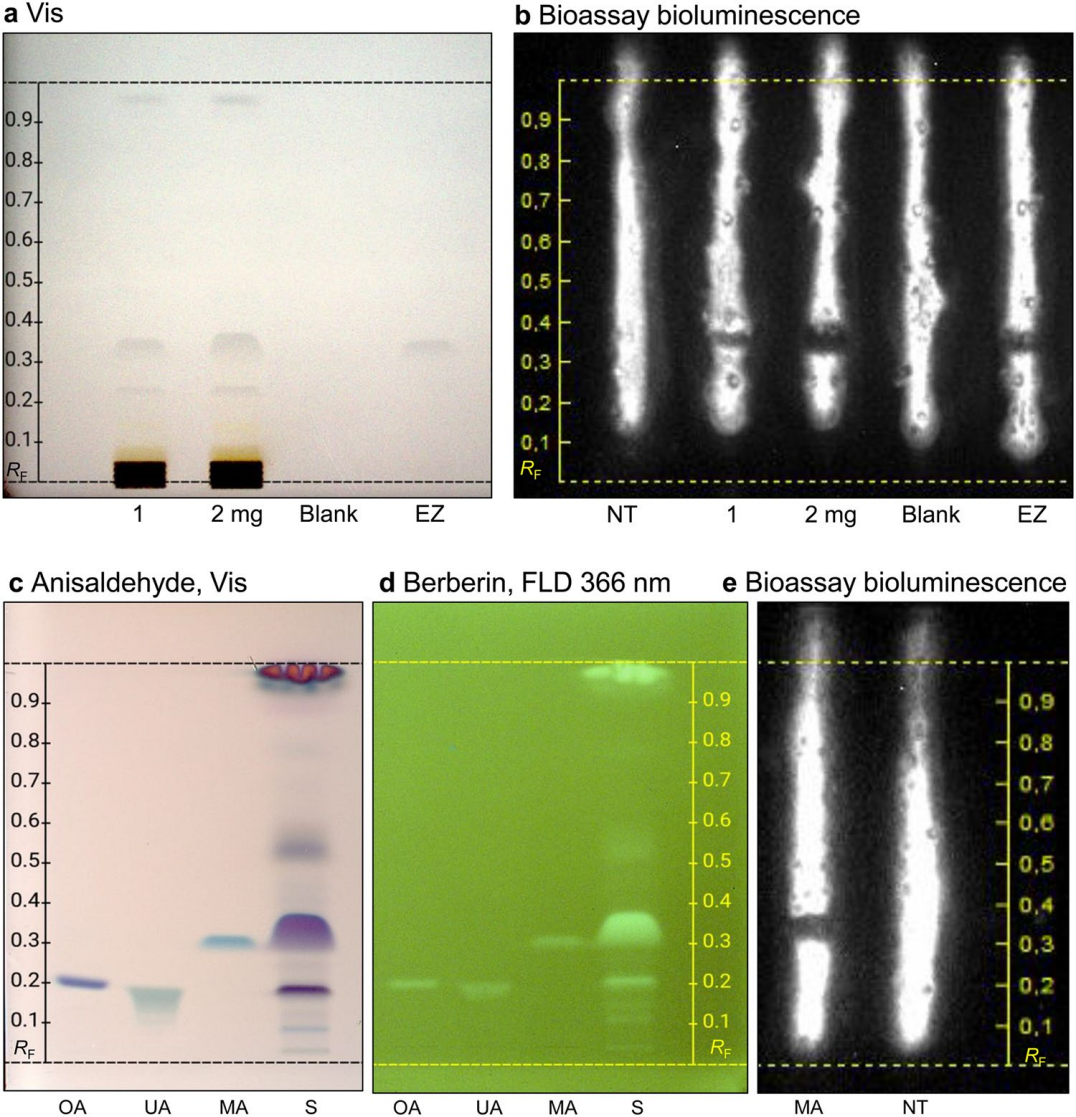
Cytotoxicity comparison of pink pepper ID 4 *n-*hexane extract (10 and 20 µL, 1 and 2 mg), the eluted cytotoxic substance zone (EZ, 100 µL), and the eluted blank plate background (100 µL), analyzed as in Fig. 3, and detected under white light illumination (**a**) and via the bioluminescence reduction after incubation with HEK 293 T-CMV-ELuc reporter cells for 24 h (**b**), as well as of (**c**) oleanolic acid (OA), ursolic acid (UA), and moronic acid (MA), 5 µg/band each, detected either at white light illumination using the *p*-anisaldehyde sulfuric acid reagent or (**d**) at FLD 366 nm using the berberine reagent, and (**e**) via the bioluminescence reduction after incubation with HEK 293 T-CMV-ELuc reporter cells for 24 h (MA, 10 µg/band; NT: not treated cells used as negative control)

Reporter cell incubation was repeated to compare the heart-cut eluted zone with the *n*-hexane sample extract, which corroborated the cytotoxic activity of the eluted zone (Fig. 6a, b). To compare *hR*_F_ values of known triterpenoic acids with the cytotoxic substance zone, those with similar molecular mass and commercially available standards were applied to the RP-HPTLC plate and analyzed in parallel to the *n*-hexane extract (sample ID 4) using *n*-hexane/acetone 4/1 (*V/V*). As expected, oleanolic and ursolic acid having two additional hydrogens did not match with the molecular formula and *hR*_F_ value of the cytotoxic substance zone of interest. However, there was another prominent band with no cytotoxic activity in sample ID 4 that corresponded to oleanolic acid at *hR*_F_ 15. This result is confirmed by previous identification of oleanolic acid in *S. terebinthifolia* dichloromethane extract analyzed via APCI-HRMS (Vieira et al., 2015). Moronic acid, which was also reported to be present in the apolar extracts of pink pepper berries (Vieira et al., 2015), matched with the *hR*_F_ value and the molecular formula obtained for the cytotoxic substance zone (C_30_H_46_O_3_). This match was confirmed by derivatization with the *p-*anisaldehyde sulfuric acid reagent and berberine reagent, indicating that moronic acid is present in the cytotoxic substance zone (Fig. 6c, d). However, the color after the *p-*anisaldehyde sulfuric acid reagent differed between the sample (mainly lilac but also blue hue at the upper zone edge) and the moronic acid (blue). A similar difference in the color was observed for the steric isomers oleanolic acid (lilac; geminal methyl groups) and ursolic acid (blue; vicinal methyl groups). Hence based on the blue color, the bought moronic acid reference standard could be the steric isomer with vicinal methyl groups (instead of geminal ones) since, according to the specification, the standard purity was only measured with HPLC and corona-charged aerosol detection which can not differentiate the isomers. Further, it was assumed based on the color formation after the derivatization with the *p*-anisaldehyde sulfuric acid reagent that both isomers were present in the sample zone (lilac with blue upper edge). This isomeric issue could be studied in more detail via NMR, or via orthogonal 8D or 12D hyphenated HPTLC workflows (Schreiner & Morlock, 2021; Schreiner et al., 2021), or via sample–reference co-chromatography as overlapped application, followed by isomer-selective planar separation. Finally, the planar bioluminescent cytotoxicity bioassay was also performed for the bought moronic acid reference standard, which proved its cytotoxic activity against the HEK 293 T-CMV-﻿ELuc cell line, corroborating the identification of this substance in the cytotoxic substance zone of the pink pepper extract through APCI-HRMS (Fig. 6e).

Nevertheless, the cytotoxic substance zone might still contain other triterpenoids with the same molecular mass, which migrate together due to similar physicochemical properties. The mentioned apolar mobile phase system *n*-hexane – toluene – tetrahydrofuran 10:1:2 (*V*/*V*/*V*) separated very well the apolar sample part (Fig. S3), as observed at white light illumination after derivatization with the *p*-anisaldehyde sulfuric acid reagent, and demonstrated the sample´s richness in similar molecule structures. Also other studies have previously described methods for the identification of individual triterpenes using TLC, and have shown that the methods do not always have enough resolution to separate substances with very similar chemical structures (Martelanc et al., 2009; Naumoska & Vovk, 2015; Navarrete et al., 2006).

The presence of triterpenes with various biological activities has been confirmed for other members of the family Anacardiaceae, such as the genus *Pistacia* and *Rhus*, which are also used as food (Bozorgi et al., 2013; Brieudes et al., 2021). The promising use of plant triterpenoids and their derivatives in cancer treatment has also been extensively discussed in the last years, with the perspective to use these molecules as backbones for the production of semi-synthetic derivatives with improved activity and bioavailability (Oramas-Royo et al., 2010; Özdemir & Wimmer, 2022; Wang et al., 2022a). Oleanolic, betulinic and ursolic acids are good examples of well-studied pentacyclic triterpenes that have cytotoxic activities and selectivity against multiple cancer cell lines, such as breast, cervical, and colorectal cancer, sarcomas and in vivo models (Aswathy et al., 2022; Chan et al., 2019). Although not yet fully understood, several studies have demonstrated that pentacyclic triterpenes can induce cell death by apoptosis (Chudzik et al., 2015; Coricovac et al., 2021; Hodoň et al., 2022; Mioc et al., 2022; Oprean et al., 2018; Ren & Kinghorn, 2019) or autophagy (El-Baba et al., 2021; Fogde et al., 2022; Wang et al., 2022b). Also, moronic acid was shown to have cytotoxic properties (Rios et al., 2001) as well as other beneficial effects such as anti-viral (Chang et al., 2010; Yu et al., 2006), anti-inflammatory (Ruan & Zha, 2022) and anti-diabetic activity (Cerón-Romero et al., 2016; Estrada-Soto et al., 2022; Ramírez-Espinosa et al., 2013), indicating the potential of triterpenoic acids and the necessity of further studies for the investigation of their biological properties.

## Conclusions

The developed hyphenated RP-HPTLC–UV/Vis/FLD–bioluminescent cytotoxicity bioassay–FIA–APCI-HRMS method provided straightforward bioprofiling of 11 pink pepper samples. Therein, a newly detected cytotoxic substance zone was tentatively assigned to be the pentacyclic triterpenoic moronic acid, identifying a potential anti-cancer agent promising for downstream mechanistic studies. Although the cytotoxicity of moronic acid was already known, it was not known for *Schinus* ssp. fruit samples. Based on the new findings, it is necessary to clarify in more detail what it means to season with pink pepper. What quantity is usually ingested, what is the resulting cytotoxic effect during the oral contact, and what is the influence of the food matrix or the preparation of the food on the cytotoxic effect. Recommendations on how best to use pink pepper can then be adapted to ensure consumer safety (cytotoxicity might be highest when adding pink pepper at the end of the dish). The comparatively faster and cheaper workflow for dereplication was proven for the discovery of a cytotoxic compound not in the previous focus detected in a frequently used spice. Due to the sustainability of the non-targeted hyphenated HPTLC methodology, it is assumed to substitute commonly used bio-guided fractionation in the future.

## Supplementary Information

Below is the link to the electronic supplementary material.

Supplementary file1 (PDF 967 KB)

## Data Availability

Data will be made available upon request.
