# Supplementary material for: Chemical and cytotoxicity profiles of 11 pink pepper (Schinus spp.) samples via non-targeted hyphenated high-performance thin-layer chromatography
Source: Metabolomics. 2023 May 2;19(5):48. doi: 10.1007/s11306-023-02008-8 (PMC10154279; doi:10.1007/s11306-023-02008-8)
Supplement: Supplementary file 1 — Supplementary file1 (PDF 967 KB) [file 11306_2023_2008_MOESM1_ESM.pdf]

Supplementary Materials

Chemical and cytotoxicity profiles of 11 pink pepper (*Schinus* spp.) samples via non-targeted hyphenated high-performance thin-layer chromatography

Fernanda L. B. Mügge, Gertrud E. Morlock*

Chair of Food Science, Institute of Nutritional Science, and Interdisciplinary Research Center, Justus Liebig University Giessen, Heinrich-Buff-Ring 26–32, 35392 Giessen, Germany.

*Corresponding author
Email: gertrud.morlock@uni-giessen.de
Tel. +49 641 99 39140

Table S1 Investigated pink pepper samples obtained from German supermarkets or online stores

**ID**	**Product sample**	**Brand**	**Listed species**	**Country of origin**
1	Rosa Pfeffer	Hanse&Pepper Gewürzkontor (Reinbek, Germany)	Not declared	Not declared
2	Rosa Pfeffer	Alpi Nature (Sofia, Bulgaria)	Not declared	Brazil
3	Pfeffer Rosa	Blank's/vom Achterhof (Uplengen, Germany)	Not declared	Brazil
4	Rosa Pfeffer	Valley of tea (Brügge, Belgium)	*S. molle*	Brazil
5	Roter Pfeffer	Bremer Gewürzhandel (Bremen, Germany)	*S. terebinthifolia*	Not declared
6	Rosa Pfefferbeeren	Concidea (Gröbenzell, Germany)	*Schinus*	Brazil
7	Rosa Beeren	BenCondito (Vienna, Austria)	*S. molle*	Not declared
8	Rosa Pfeffer	Ankerkraut (Hamburg, Germany)	*Schinus*	Not declared
9	Rosa Beeren	Gerüche Küche (Lohmar, Germany)	*S. terebinthifolia*	Brazil
10	Rosa Beeren	Fuchs (Dissen, Germany)	*S. terebinthifolia*	Not declared
11	Rosa Beeren	Fuchs (Dissen, Germany)	*S. terebinthifolia*	Not declared

Table S2 Mobile phase development exemplarily shown for sample ID 1 extracted with ethyl acetate – ethanol – water 1:1:1, applied (10 µL/band, 1 mg/band) on the RP-18 W HPTLC plate, separated with the respective mobile phase system up to 7 cm and detected at FLD 366 nm

**Mobile phase**	**FLD 366 nm**
Methanol – acetonitrile – formic acid<br>1.75:1.75:0.1	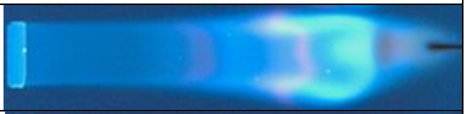
Toluene – ethyl acetate – formic acid – isopropanol<br>1:1.25:0.1:0.25	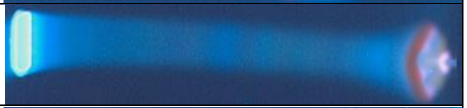
Chloroform – acetone – acetic acid – methanol<br>3:0.75:0.1:0.2	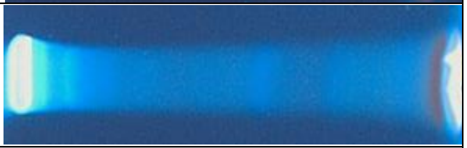
Toluene – acetic acid<br>2.5:0.1	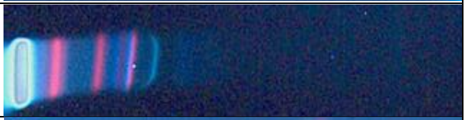
Toluene – ethyl acetate – formic acid – methanol<br>1.875:0.63:0.15:0.255	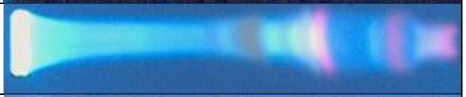
Toluene – acetone – acetic acid – methanol<br>3:0.5:0.1:0.1	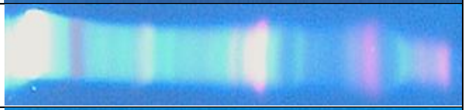
Toluene – acetonitrile – formic acid – methanol<br>3:0.5:0.1:0.2	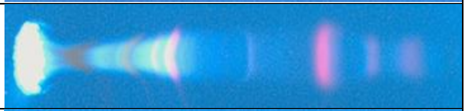
Toluene – acetone – formic acid – methanol<br>3:0.5:0.1:0.2	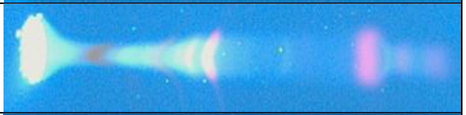
Toluene – acetone – acetic acid – methanol<br>3:0.5:0.1:0.1	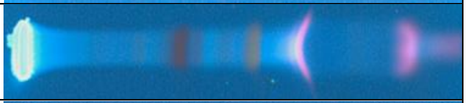
Toluene – chloroform – ethyl acetate – acetic acid<br>1:1.2:0.2:0.05	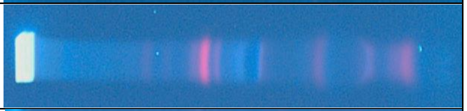
Toluene – chloroform – ethyl acetate – acetic acid<br>1:1:0.2:0.05	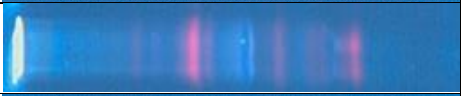
Toluene – ethyl acetate – acetic acid – methanol<br>3:0.5:0.1:0.2	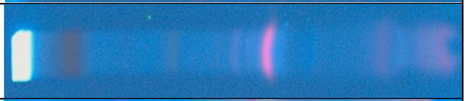
Toluene – ethyl acetate – acetic acid – methanol<br>3:0.5:0.1:0.2<br>here, after derivatization with anisaldehyde-sulfuric acid reagent	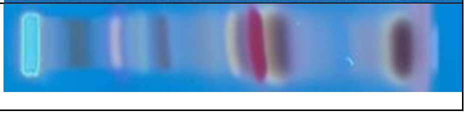

Table S3 Instrumental settings for FIA–APCI$^{+/–}$-HRMS recording of cytotoxic substance zones eluted from the RP-18 W HPTLC plate

**FIA**	
Eluent	Methanol
Flow rate	0.2 mL/min
Injection volume	4 µL
**APCI$^{+/–}$probe**	
Spray voltage	5 kV
Capillary temperature	250 ˚C
Sheath gas	15
Aux gas	5
Sweep gas	0
Maximum spary current	80 mA (+), 10 mA (-)
S-Lens RF level	50
**HRMS full scan**	
Resolution	280.000
AGC target	1e6
Maximum inject time	250 ms
Scan range	*m*/*z* 100-1000
Spectrum data type	Profile
Lock masses	Positive ionization mode:<br>413.26623 [diisooctylphthalate+Na]$^{+}$<br>301.14103 [dibutylphthalate+Na]$^{+}$
	Negative ionization mode:<br>112.98563 [formic acid sodium dimer−Na]$^{–}$

Table S4 APCI$^{-/+}$-HRMS fragmentation signals from the cytotoxic substance zone eluted from the RP-18 W HPTLC plate (Fig. S4), all tentatively assigned to moronic acid ($C_{30}H_{46}O_3$), which is a triterpenoic acid

**Measured signal *m/z***	**Signal assignment**	**Mass error Δ ppm**	**Molecular formula**
453.3378	$[M-H]^-$	0.37	$C_{30}H_{46}O_3$
907.6831	$[2M-H]^-$	0.48	
455.3517	$[M+H]^+$	0.60	
437.3413	$[M-H_2O+H]^+$	0.24	
469.3673	$[M+CH_2+H]^+$	0.68	
409.3463	$[M-CH_2O_2+H]^+$	0.47	
909.6969	$[2M+H]^+$	0.26	

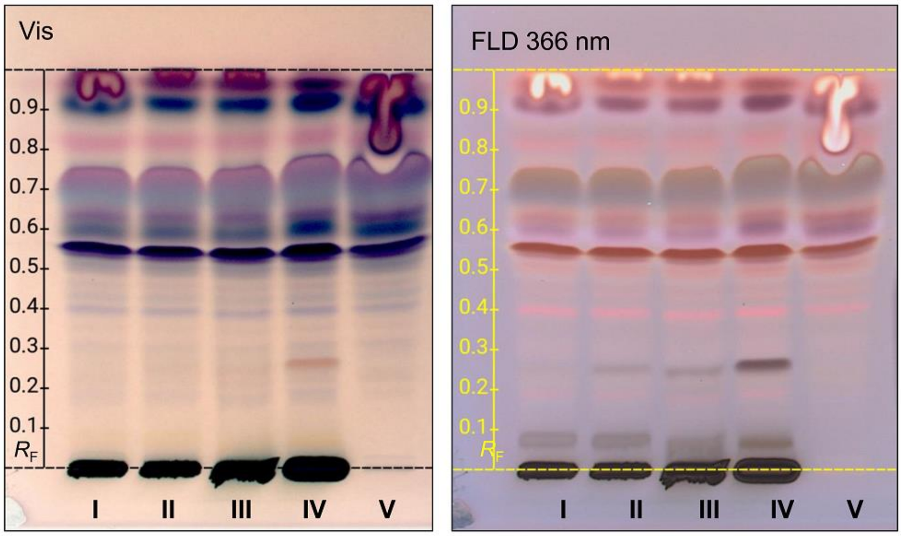

Fig. S1 Comparison of the physicochemical profiles of pink pepper sample ID 1 (Table S1) extracted with different extractants (100 mg/mL each), *i.e.* (**I**) methanol, (**II**) ethanol, (**III**) ethanol – water 9:1 (*V*/*V*), (**IV**) ethyl acetate – ethanol – water 1:1:1 (*V*/*V*/*V*), and (**V**) *n*-hexane. Each extract was applied (10 µL/band, representing maximal 1 mg extracted sample) on the RP-18 W HPTLC plate as 7 mm band, separated using toluene – ethyl acetate – methanol – acetic acid 30:5:2:1 (*V*/*V*/*V*/*V*) up to 7 cm and detected after derivatization with anisaldehyde sulfuric acid reagent under white light illumination (Vis) and FLD 366 nm.

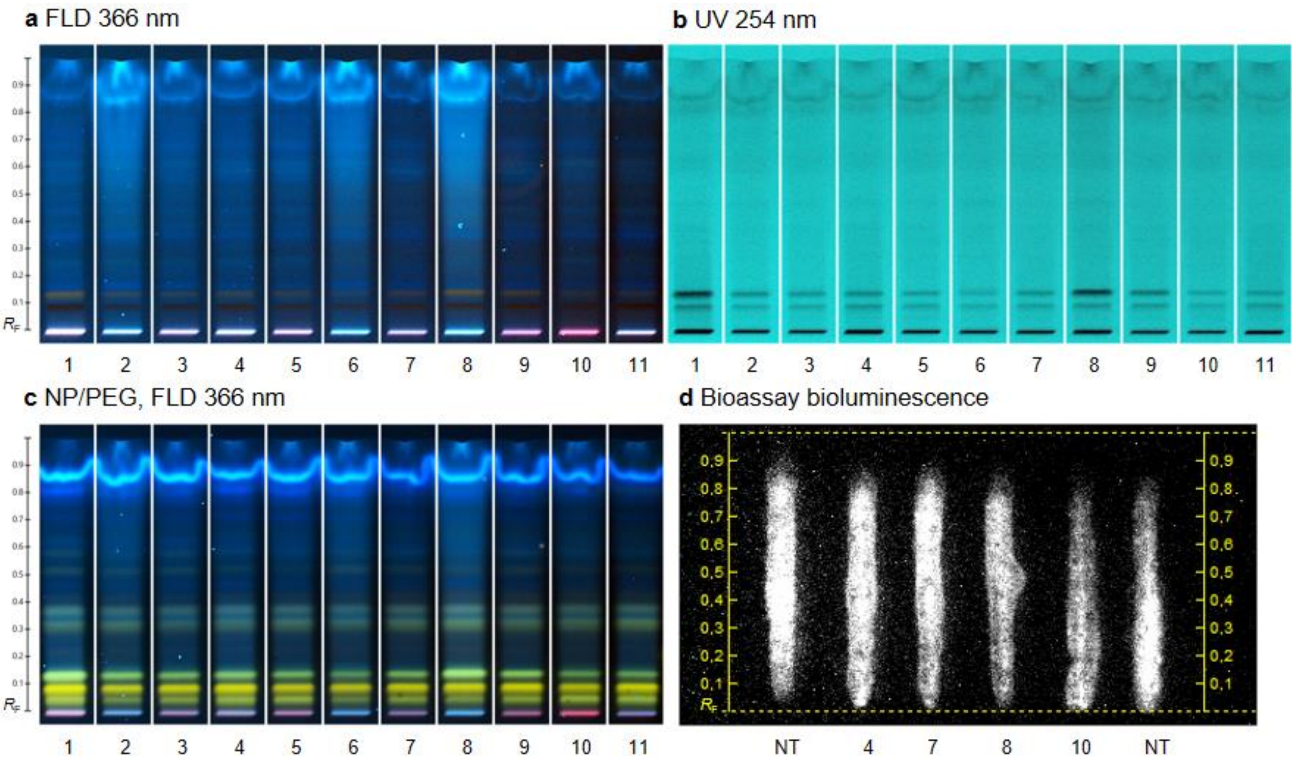

Fig. S2 Comparison of the polar physicochemical profiles of the ethyl acetate – water – ethanol 1/1/1 (*V/V/V*) extracts of eleven pink pepper samples (100 mg/mL, 10 µL/band) applied as 7-mm band each on the RP-18 W HPTLC plate, separated with water – acetonitrile – methanol – tetrahydrofuran – formic acid 15/5/5/1/1 (*V/V/V/V/V*) up to 7 cm, and detected at (**a**) FLD 366 nm, (**b**) UV 254 nm, and (**c**) FLD 366 nm after derivatization with natural product A reagent and polyethylene glycol 6000 (NP/PEG). (**d**) Cytotoxicity profiles of four selected pink pepper extract samples after the planar cytotoxicity bioassay (HEK 293T-CMV-Eluc reporter cell suspension of 5000 cells/µL, 400 µL applied per stripe, incubated for 24 h, dried for 5 min under cold air, immersed in lysis buffer containing D-luciferin; NT: not treated cells used as the negative control) detected via the bioluminescence depicted as greyscale image.

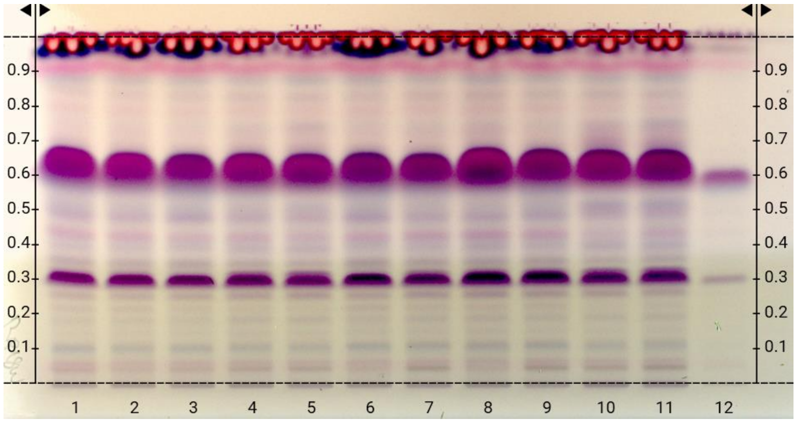

Fig. S3 Comparison of the apolar physicochemical profiles of the eleven *n*-hexane extracts (100 mg/mL, 20 µL/band each) of pink pepper (tracks 1–11) in comparison to ID 1 seed extract (track 12) applied as 8-mm band each on the RP-18 W HPTLC plate separated with *n*-hexane – toluene – tetrahydrofuran 10:1:2 (*V/V/V;* relative humidity 63%) up to 7 cm and detected under white light illumination after derivatization with the anisaldehyde sulfuric acid reagent.

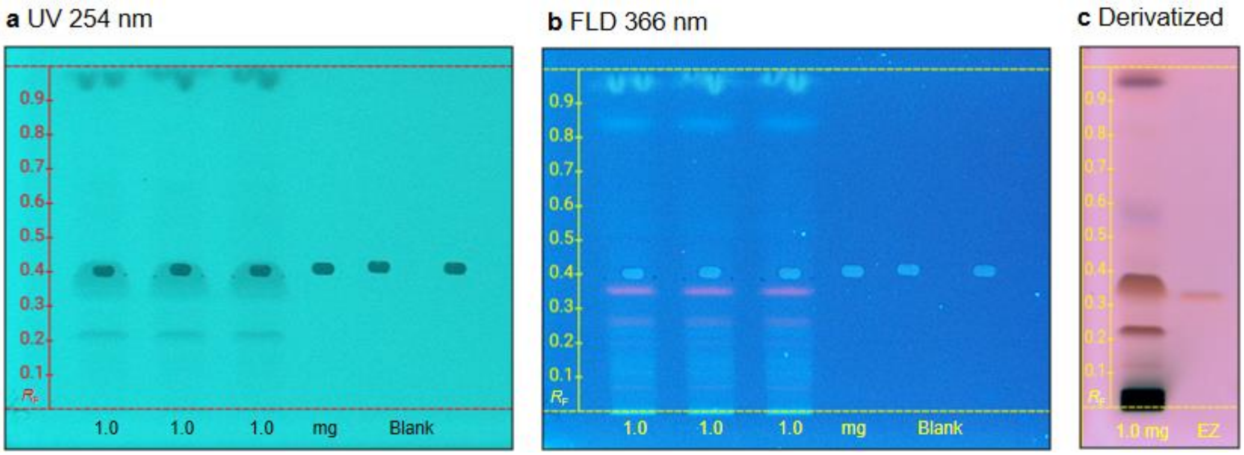

Fig. S4 Elution head imprints documented at (**a**) UV 254 nm and (**b**) FLD 366 nm after the FIA–APCI-HRMS recording of the cytotoxic zone of interest (marked at UV 254 nm as observed in the RP-HPTLC-chromatogram in Fig. 3 and cytotoxicity bioautogram in Fig. 4) in the *n*-hexane extract of the pink pepper sample ID 4 (100 mg/mL, 10 µL/band) applied as 7-mm band in triplicate, separated using *n*-hexane – acetone 4:1 (*V/V*), and eluted in triplicate with ethyl acetate from the RP-18 W HPTLC plate via the autoTLC–LC–MS interface (Mehl et al. 2021) into the same sampler vial (for comparison, three plate background elutions were also pooled as blank). (**c**) After evaporation of the ethyl acetate in the pooled eluted zones and reconstitution in methanol, the eluted zone (EZ, 50 µL/band) was co-applied, separated as mentioned, and detected at FLD 366 nm after derivatization with anisaldehyde sulfuric acid reagent.
